# 基于共价有机骨架材料的磁固相萃取-超高效液相色谱-串联质谱法测定水中4种杀菌剂

**DOI:** 10.3724/SP.J.1123.2022.08023

**Published:** 2022-11-08

**Authors:** Pan WANG, Jiping MA, Shuang LI, Jiawen CHENG, Zongyue ZOU

**Affiliations:** 青岛理工大学环境与市政工程学院, 山东 青岛 266520; School of Environmental and Municipal Engineering, Qingdao University of Technology, Qingdao 266520, China

**Keywords:** 超高效液相色谱-串联质谱, 磁固相萃取, 共价有机骨架材料, 杀菌剂, 环境水体, ultrahigh performance liquid chromatography-tandem mass spectrometry (UHPLC-MS/MS), magnetic solid phase extraction (MSPE), covalent organic frameworks (COFs), fungicide, environmental waters

## Abstract

杀菌剂在环境中长期富集后会引起土壤和植物病害,并能借助雨水或灌溉渗透到深层土壤和地下水中,威胁水体环境和人体健康。因此针对水中杀菌剂开发简单快速、高效灵敏的分析方法至关重要。该研究通过原位合成法制备了磁性共价有机骨架材料Fe3O4@TpBD,将其作为萃取吸附剂,富集环境水体中苯并咪唑杀菌剂(噻菌灵、麦穗宁、多菌灵)和有机硫杀菌剂(稻瘟灵)。利用Fe3O4@TpBD与杀菌剂之间的*π-π*共轭、氢键和静电作用进行吸附,结合超高效液相色谱-串联质谱法(UHPLC-MS/MS)进行检测,建立了测定水中4种痕量杀菌剂的分析方法。通过透射电子显微镜、X射线衍射及傅里叶变换红外光谱等方式对Fe3O4@TpBD进行表征,以证明材料的成功合成。对萃取条件进行一系列的优化(Fe3O4@TpBD的磁性比例及用量、水样pH、吸附时间、洗脱液的种类及体积、洗脱时间、NaCl含量),确定了最佳萃取条件。4种杀菌剂在3~1200 ng/L的范围内线性关系良好,线性相关系数均大于0.998,方法的检出限和定量限分别为0.06~0.28 ng/L和0.20~0.92 ng/L。在15、150和600 ng/L 3个加标水平下进行加标回收试验,日内和日间精密度分别为2.8%~10.0%和4.4%~15.7%。将该方法用于实际水样的检测,4种杀菌剂的加标回收率为77.1%~119.1%,在水库水中检测出多菌灵,含量为27.5 ng/L。该方法灵敏度高,准确度和精密度良好,操作简单,耗时短。

## Abstract (en)

Fungicides can lead to soil and plant diseases after long-term enrichment in the environment; they can also penetrate deeper into the soil and groundwater by rainwater or irrigation, threatening the water environment and human health. Therefore, it is crucial to develop a simple, rapid, efficient, and sensitive analytical method for the detection of fungicides in the water environment. Sample pretreatment is important for the extraction and enrichment of pollutants from environmental water. Magnetic solid phase extraction (MSPE) is a new sample pretreatment method, which uses magnetic materials as adsorbents dispersed in solution, and rapid separation can be achieved by the aid of external magnets. Because of its advantages of short analytical time, less organic solvent consumption, and easy separation of adsorbents, MSPE has attracted much attention. The key to MSPE is the preparation of highly selective magnetic adsorbents. Covalent organic frameworks have the advantages of large surface area, good chemical and thermal stability, tunable porous structure, low density, and easy functionalization, all of which are ideal for adsorbing fungicides. The concentration of fungicides in environmental water is low. Ultrahigh performance liquid chromatography-tandem mass spectrometry (UHPLC-MS/MS) has high sensitivity and high selectivity, which is suitable for the analysis of fungicides. In this work, a magnetic covalent organic framework Fe3O4@TpBD was prepared by an *in situ* method, as the MSPE sorbent material to enrich of benzimidazole fungicides (thiabendazole, carbendazim, fuberidazole) and organic sulfur fungicide (isoprothiolane) in environmental water. An extraction method based on *π-π* conjugation, hydrogen bonding, and electrostatic interaction between Fe3O4@TpBD and the fungicides, in combination with UHPLC-MS/MS, was developed for the determination of four trace fungicides in water. Transmission electron microscopy (TEM), X-ray diffractometry (XRD), and Fourier transform-infrared spectroscopy (FT-IR) were performed to confirm the successful synthesis of Fe3O4@TpBD and to characterize this material. A series of experiments were carried out to decide the optimal extraction conditions, i. e., the magnetic ratio and dosage of Fe3O4@TpBD, pH of the water sample, adsorption time, type and volume of the eluent, elution time, and salinity. Gradient elution was carried out with methanol-water as the mobile phase. The target analytes were separated on an ACQUITY UPLC BEH C18 column (100 mm×2.1 mm, 1.7 μm), and multiple reaction monitoring (MRM) was conducted in the positive electrospray ionization mode. The ion source temperature and ion source voltage were set to 500 ℃ and 5 kV, respectively. The analytical method was established under the optimized extraction conditions. The four fungicides showed good linearity in the range of 3-1200 ng/L, with linear correlation coefficients greater than 0.998. The limits of detection (LODs) and limits of quantification (LOQs) of this developed method were 0.06-0.28 ng/L and 0.20-0.92 ng/L, respectively. Recovery tests were performed at three spiked levels of 15, 150, and 600 ng/L, with relative standard deviations of 2.8% to 10.0% (intra-day) and 4.4% to 15.7% (inter-day). The accuracy of the established analytical method was investigated by using it to test real water samples, and satisfactory recoveries for the four analytes were achieved within 77.1% to 119.1%. Trace amounts of carbendazim were detected in the reservoir water at 27.5 ng/L. The method has good sensitivity, accuracy, and precision, and the operation process is convenient.

新污染物是指近年来被发现或受到关注的,对生态环境或人体健康存在风险,未纳入管理或现有管理措施不能有效防控其风险的污染物^[1]^。杀菌剂是新污染物之一,部分种类的杀菌剂被列入新版《生活饮用水卫生标准》中。噻菌灵(thiabendazole, TBZ)、麦穗宁(fuberidazole, FBZ)和多菌灵(carbendazim, MBC)是以苯并咪唑环为母体的杀菌剂,应用于水果等多种作物真菌性病害的防治。稻瘟灵(isoprothiolane, IPT)属于有机硫杀菌剂,用于预防和控制水稻稻瘟病。这些化合物在环境中经过长期富集后不但会引起土壤和植物体的病害问题,还会在雨水或灌溉的促进下渗透到更深层的土壤和地下水中,威胁水体环境和人体健康,因此针对水中杀菌剂开发高效灵敏的分析方法十分重要。由于苯并咪唑杀菌剂具有热不稳定性,用气相色谱分析时往往需要衍生化^[2]^,而液相色谱无需繁琐的衍生过程,且分离性能好、灵敏度高,因此高效液相色谱法(HPLC)^[3,4]^、液相色谱-串联质谱法(LC-MS/MS)^[5]^更适用于其分析。

杀菌剂在环境水体中含量低且水样基质复杂,在仪器分析之前需采用合适的样品前处理技术。常用的样品前处理方法有固相萃取(solid phase extraction, SPE)^[6]^、固相微萃取(solid phase microextraction, SPME)^[7]^、分散固相萃取(dispersive solid phase extraction, DSPE)^[8]^等。SPE是最常用的样品前处理技术,具有回收率高、有机溶剂用量少、易于自动化操作等优点,但存在样品前处理时间长、固相萃取小柱易堵塞以及商品化固相萃取小柱对于某些目标化合物选择性差等问题。DSPE操作简便、萃取时间短,但对于纳米材料吸附剂存在不易回收等问题。近年来,研究者开发了磁固相萃取(magnetic solid phase extraction, MSPE)^[9]^、分散膜固相萃取(dispersive membrane extraction, DME)^[10]^等方法以解决上述问题。MSPE是一种新型SPE技术,使用磁性材料作为吸附剂分散在溶液中,然后通过外部磁铁来实现快速分离。与传统SPE技术相比,MSPE具有许多明显优点,包括耗时短、有机溶剂消耗量少以及吸附剂易于分离等。而MSPE的关键在于制备高选择性的磁性吸附剂,我们课题组已制备了磁性多壁碳纳米管(MWCNTs)、磁性MOF-5、磁性TpBD、磁性MIL-101等磁性吸附剂^[11⇓⇓-14]^用于环境水样中多环芳烃、农药及消毒副产物的富集和分析。

共价有机骨架材料(covalent organic frameworks, COFs)是由C、B、O、Si、N等轻元素通过共价键结合而成的有机二维或三维多孔晶体结构的新型材料^[15]^。与传统的多孔晶体材料相比,COFs具有比表面积大、化学稳定性和热稳定性好、多孔结构可调、密度低、易于功能化等优点。由于其高吸附能力和结构可调性,COFs是吸附有机小分子^[16]^和小离子的理想选择^[17]^。与其他吸附剂材料相比,COFs因其强共价相互作用具有更稳定的结构,*π-π*相互作用提高了COFs与目标物相互作用的亲和力^[18]^。基于这些优点,COFs在样品前处理方面得到广泛应用。Xu等^[19]^以三(4-氨基苯基)胺(TAPA)和三(4-甲酰基苯基)苯(TFPB)合成的TAPA-TFPB-COFs作为固相萃取柱填料富集水和食品样品中的喹诺酮类抗生素。Wu等^[20]^将TpBD用作SPME纤维涂层,吸附富集蜂蜜和黄桃中的7种氯酚。

本文采用原位合成法将三醛基间苯三酚(Tp)和联苯胺(BD)合成的TpBD包覆于Fe_3_O_4_外部,制备得到核壳型磁性材料Fe_3_O_4_@TpBD,并对其采用透射电子显微镜(TEM)、X射线衍射(XRD)和傅里叶变换红外光谱(FT-IR)等方式进行表征,优化了影响MSPE效果的主要因素。将涡旋辅助MSPE与UHPLC-MS/MS相结合,在最佳萃取条件下建立了一种快速、灵敏、易操作的分析方法并应用于水中痕量杀菌剂的富集分析。

## 1 实验部分

### 1.1 仪器与试剂

QTRAP 3500超高效液相色谱-三重四极杆质谱仪(美国AB Sciex公司), WB100-1恒温数显水浴锅(湖州市群安实验仪器有限公司), XW-18D+漩涡混合器(绍兴市苏珀仪器有限公司), Millipore D-24UV超纯水机(美国Millipore公司), JEM-2100 F电子透射显微镜(日本JEOL公司); SmartLab9K X射线衍射仪(日本Rigaku公司); Frontier傅里叶变换红外光谱仪(美国PerkinElmer公司); Lakeshore-7404振动样品磁强计(美国Lakeshore公司); ASAP2460表面积及孔径分析仪(美国Micromeritics公司); ZetaPlus电位仪(美国Brookhaven公司)。

杀菌剂标准品:多菌灵(纯度≥99.9%)和噻菌灵(纯度≥99.9%)购自广州丹安仪器仪表有限公司,麦穗宁(纯度≥99.7%)购自天津阿尔塔科技有限公司,稻瘟灵(纯度98%)购自上海贤鼎生物科技有限公司,结构与性质见表1。色谱级甲醇和乙腈购自德国默克公司,氨水购自国药集团化学试剂有限公司,Fe_3_O_4_ (200 nm)购自上海麦克林生化科技有限公司,联苯胺(纯度95%)购自上海阿拉丁生化科技股份有限公司,三醛基间苯三酚(纯度97%)购自吉林中科研伸科技有限公司,分析纯甲醇和四氢呋喃(THF)购自天津市北辰方正试剂厂。

**表1:** 4种杀菌剂的结构与性质

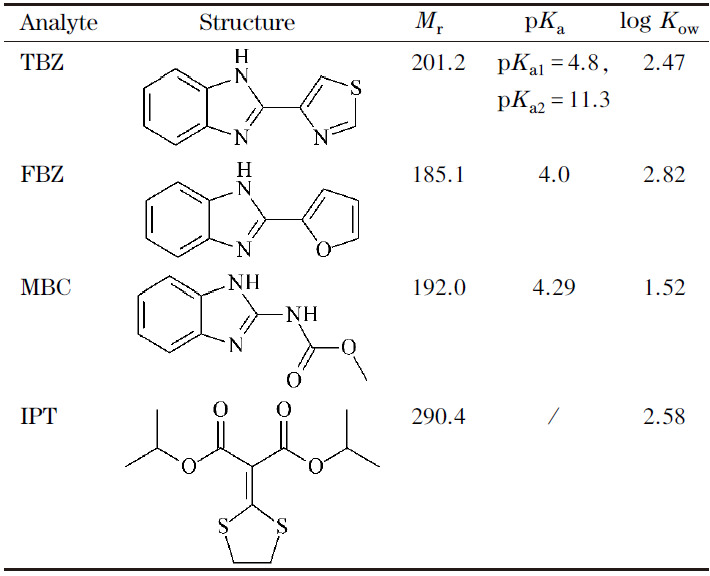

TBZ: thiabendazole; FBZ: fuberidazole; MBC: carbendazim; IPT: isoprothiolane. /: not available.

实际水样采集自青岛丁家河水库和实验室自来水,所有水样均用0.45 μm滤膜过滤,并在4 ℃下保存于棕色玻璃瓶中。

### 1.2 材料制备

Fe_3_O_4_@TpBD的合成是在文献^[13]^的基础上进行的,如图1所示。先将48 mg Fe_3_O_4_分散在11 mL THF中超声混匀,再加入24 mg BD继续超声10 min,然后在50 ℃下搅拌回流50 min。将含有18 mg TP的4 mL THF溶液逐滴加入上述混合物中机械搅拌3 h,将获得的棕色材料用磁铁分离,用甲醇洗涤3次后在60 ℃下真空干燥。

**图1:**
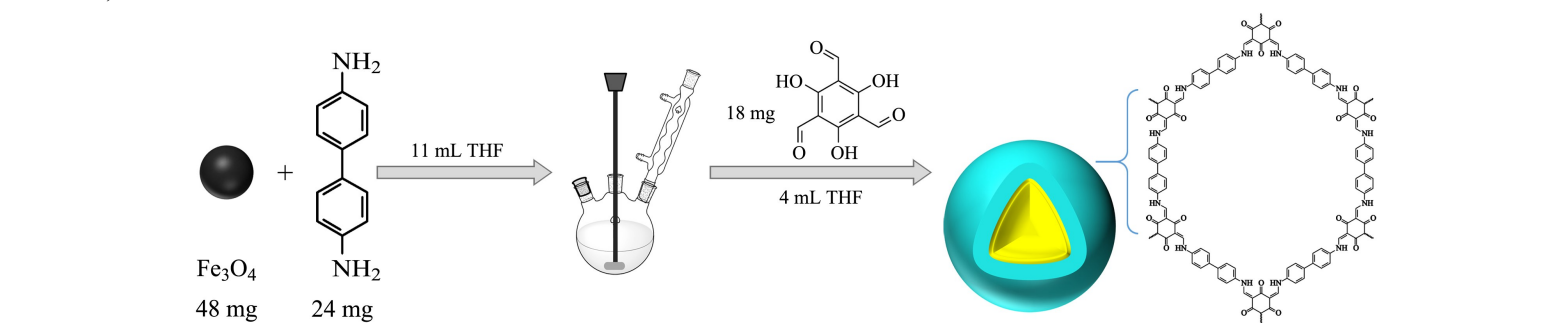
Fe_3_O_4_@TpBD的制备过程

### 1.3 样品前处理

磁固相萃取步骤如图2所示,取14 mg Fe_3_O_4_@TpBD磁性材料于50 mL离心管中,加入50 mL水样,涡旋4 min将Fe_3_O_4_@TpBD均匀地分散在溶液中以吸附目标化合物。萃取完成后将磁铁附着在离心管外部对Fe_3_O_4_@TpBD和水样进行分离,倒出水溶液并加入1.5 mL含3%(v/v)氨水的乙腈溶液涡旋1 min进行洗脱,过0.22 μm滤膜后用UHPLC-MS/MS进行分析。

**图2:**
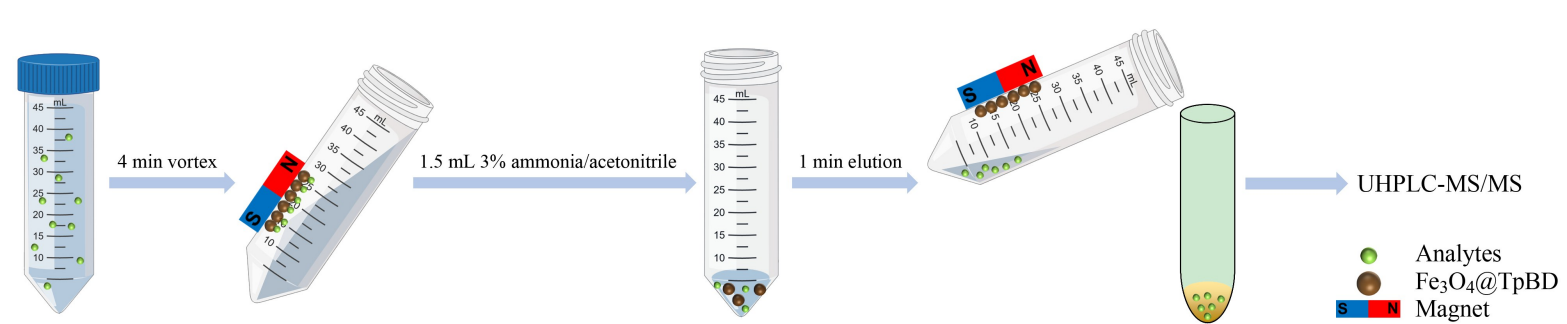
磁固相萃取步骤

### 1.4 仪器条件

色谱条件:采用ACQUITY UHPLC BEH C18色谱柱(100 mm×2.1 mm, 1.7 μm;美国Waters公司);柱温40 ℃;进样量为5 μL;流动相分别为水(A)和甲醇(B),流速0.3 mL/min。梯度洗脱程序:0~2.0 min, 70%A; 2.0~6.0 min, 70%A~10%A; 6.0~9.0 min, 10%A; 9.0~9.1 min, 10%A~70%A; 9.1~11.0 min, 70%A。

质谱条件:ESI源,正离子模式;多反应监测模式;离子源温度:500 ℃;离子源电压:5000 V;气帘气压力:2.07×10^5^ Pa;雾化气压力:3.45×10^5^ Pa;辅助器压力:4.14×10^5^ Pa。4种杀菌剂的其他质谱参数见表2。

**表2:** 4种杀菌剂的质谱参数

Analyte	t_R_/min	Precursor ion (m/z)	Product ions (m/z)	Declustering potentials/V	Collision energies/eV
TBZ	5.36	201.2	175.0^*^, 131.1	72, 72	33, 45
FBZ	5.55	185.1	156.1^*^, 92.1	84, 84	38, 43
MBC	4.84	192.0	160.0^*^, 132.8	59, 68	28, 44
IPT	6.60	290.4	189.0^*^, 231.0	46, 46	17, 31

* Quantitive ion.

## 2 结果与讨论

### 2.1 材料表征

使用TEM对Fe_3_O_4_@TpBD进行表征,图3a显示了通过TEM观察到的Fe_3_O_4_@TpBD的微观形貌。Fe_3_O_4_呈均匀的球形,TpBD附着在其表面形成了核壳结构。

**图3:**
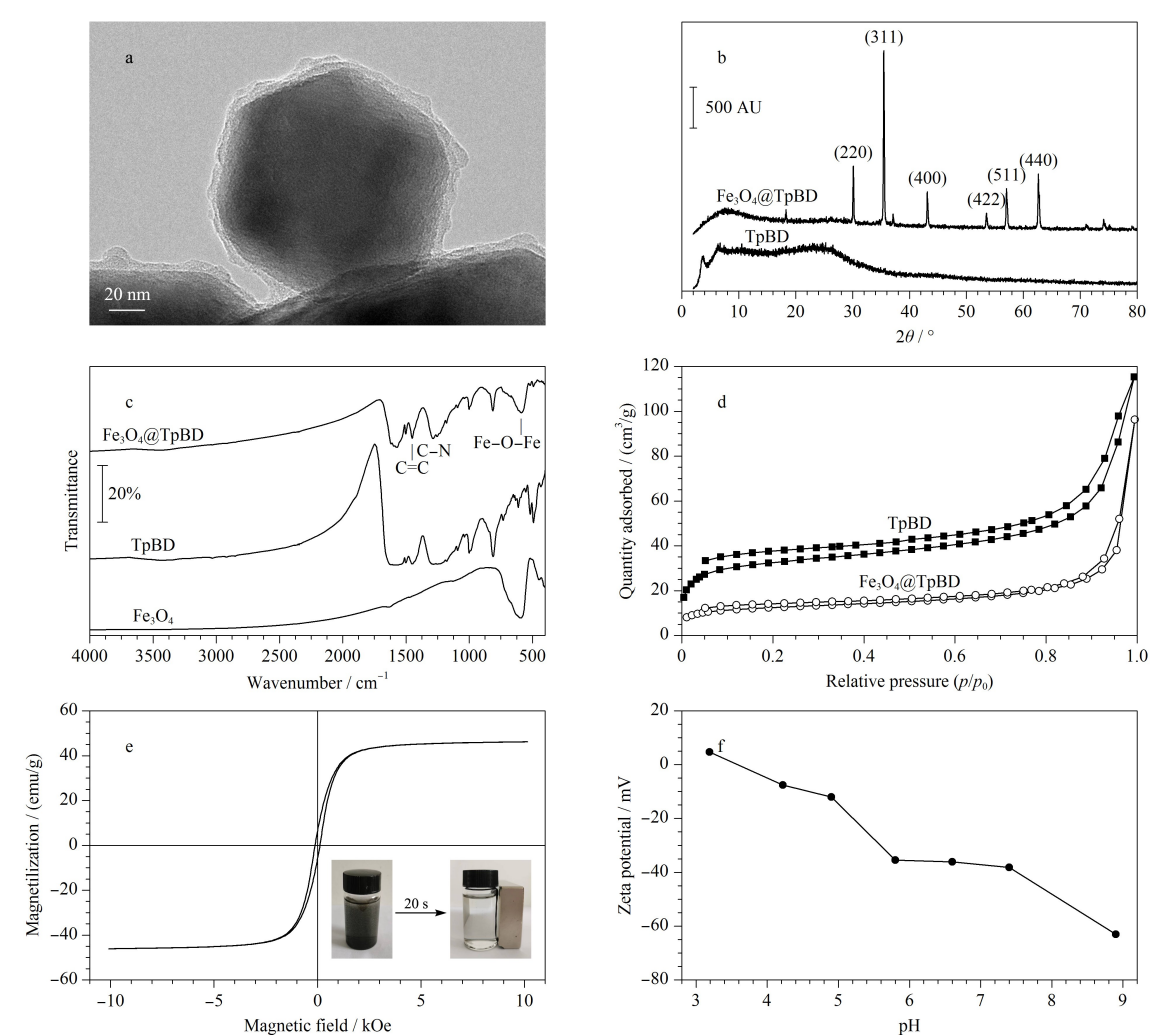
(a)Fe_3_O_4_@TpBD的透射电镜图、(b)Fe_3_O_4_@TpBD和TpBD的X射线衍射图、(c)Fe_3_O_4_、TpBD和Fe_3_O_4_@TpBD的FT-IR图、(d)TpBD和Fe_3_O_4_@TpBD的N_2_吸附-解吸等温线、(e)Fe_3_O_4_@TpBD的磁滞回线图、(f)Fe_3_O_4_@TpBD的Zeta电位图

图3b显示了Fe_3_O_4_@TpBD和TpBD的XRD图。在10~80°的2*θ*区域内,Fe_3_O_4_@TpBD中的30.1°、35.5°、43.1°、53.5°、57.0°、62.6°处的衍射峰分别是(220)、(311)、(400)、(422)、(511)、(440),对应了Fe_3_O_4_的晶体结构,与文献^[21]^报道一致。TpBD中2*θ*=3.8°处出现的微弱衍射峰说明了TpBD的结晶度较低,另外Fe_3_O_4_@TpBD中TpBD含量较少,因此衍射峰不明显,Fe_3_O_4_@TpBD在6.4°和18.2°处出现的衍射峰与文献^[22]^报道的数据相同,证明了材料的成功合成。

图3c为Fe_3_O_4_@TpBD、TpBD和Fe_3_O_4_的FT-IR图。TpBD在1286 cm^-1^处的吸收峰归因于C-N键的伸缩振动,1451 cm^-1^处的吸收峰由C=C键的伸缩振动产生^[23,24]^。对比TpBD的谱图可以发现, Fe_3_O_4_@TpBD在589 cm^-1^处的特征峰来源于Fe_3_O_4_中的Fe-O-Fe。Fe_3_O_4_@TpBD同时含有Fe_3_O_4_和TpBD的特征吸收峰,表明了材料的成功合成。

图3d为TpBD和Fe_3_O_4_@TpBD在77 K下的N_2_吸附-解吸等温线,显示了Ⅳ型N_2_吸附等温线,这是介孔结构的特征。Fe_3_O_4_@TpBD的表面积为40.78 m^2^/g,总孔体积为0.05 cm^3^/g,孔径为4.910 nm。4种杀菌剂的分子尺寸均小于1 nm, Fe_3_O_4_@TpBD适用于杀菌剂的富集。

Fe_3_O_4_@TpBD的磁滞回线如图3e所示,插图显示了Fe_3_O_4_@TpBD在水溶液中的分离过程,20 s即可快速分离。Fe_3_O_4_@TpBD的饱和磁化强度值为46.14 emu/g,表明了Fe_3_O_4_@TpBD具有强磁响应性,可以保证材料在外部磁场的作用下迅速实现磁分离。

Fe_3_O_4_@TpBD的Zeta电位如图3f所示,Fe_3_O_4_@TpBD的等电点在pH为3~4之间,pH>4时Fe_3_O_4_@TpBD呈电负性。

### 2.2 色谱条件的优化

比较了甲醇-水溶液和乙腈-水溶液作为流动相时目标化合物的分离效果。使用甲醇作为有机相时,4种目标化合物的分离效果和峰形较好,因此选择甲醇-水溶液作为流动相。4种杀菌剂的总离子色谱图见图4。

**图4:**
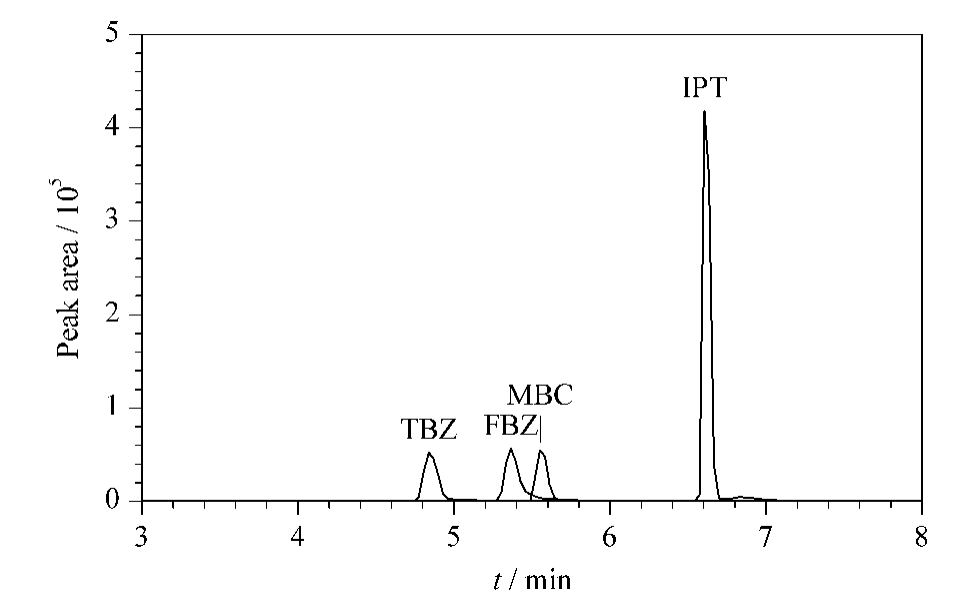
4种杀菌剂的总离子流色谱图

### 2.3 前处理条件的优化

为了获得最佳萃取效果,对Fe_3_O_4_@TpBD的磁性比例及用量、水样pH值、吸附时间、洗脱液类型及体积、洗脱时间和离子强度这些因素进行了考察,加标水样中杀菌剂的质量浓度为400 ng/L。

#### 2.3.1 磁性比例

本实验以Fe_3_O_4_@TpBD前驱体BD的量为基准,Fe_3_O_4_(8、12、24、48、72 mg)与BD(24 mg)的质量比例为磁性比例,制备了5种不同磁性比例的Fe_3_O_4_@TpBD(1:3、1:2、1:1、2:1、3:1),考察不同比例的Fe_3_O_4_@TpBD对萃取效率的影响。如图5a所示,随着Fe_3_O_4_从8 mg增加到48 mg,萃取效率上升,Fe_3_O_4_的加入增大了材料的磁性,有利于材料的快速分离,减少了目标物的损失,而BD用量太少则会降低材料对目标物的吸附量。因此选择磁性比例为2:1的Fe_3_O_4_@TpBD进行实验。

**图5:**
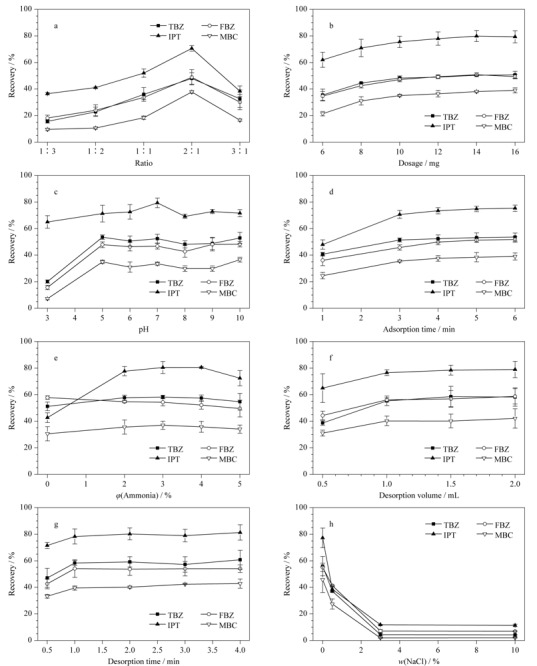
(a)Fe_3_O_4_@TpBD的磁性比例、(b)材料用量、(c)pH、(d)吸附时间、(e)洗脱液中氨水含量、(f)洗脱液体积、(g)洗脱时间和(h)NaCl含量对4种杀菌剂萃取效率的影响(n=3)

#### 2.3.2 材料用量

吸附剂的用量是影响萃取效率的关键因素,考察Fe_3_O_4_@TpBD的添加量分别为6、8、10、12、14、16 mg时的萃取效果。图5b显示,萃取效率随着Fe_3_O_4_@TpBD用量的增加逐渐提高,当Fe_3_O_4_@TpBD的用量从14 mg增加到16 mg时,萃取效率无明显上升,材料对目标物的吸附接近饱和,因此吸附剂的最佳用量为14 mg。

#### 2.3.3 水样pH值

样品溶液的pH显著影响材料对目标物的吸附效率,考察了水样pH分别为3、5、6、7、8、9、10时的萃取效果。图5c的结果表明,随着pH从3增加到5,萃取效率逐渐升高,而在5~10之间萃取效率无显著变化。这是因为苯并咪唑杀菌剂咪唑环中的两个氮原子具有酸性和碱性,使其具有质子化(p*K*_a_约5~6)或去质子化(p*K*_a_接近12)的能力^[3]^。以TBZ(p*K*_a2_=4.8, p*K*_a2_=11.3)为例,水溶液中TBZ分子中的基团可以通过质子化和去质子化形成3种不同形态。在pH<4.8时以阳离子态存在;4.8<pH<11.3时以中性分子和两性离子态存在;pH>11.3时以阴离子态存在。当溶液pH值在5~10之间时,TBZ主要以中性分子及两性离子形态存在,Fe_3_O_4_@TpBD表面均带负电荷,带负电荷的Fe_3_O_4_@TpBD和部分阳离子形态的TBZ之间存在静电吸附作用。Fe_3_O_4_@TpBD中-NH基团中的H原子作为氢键受体,与TBZ、FBZ和MBC咪唑环中的N原子以及IPT中酯基上的O原子形成氢键作用力。Fe_3_O_4_@TpBD对杀菌剂的吸附主要由*π-π*堆积和氢键相互作用以及静电吸附作用驱动,从而表现出良好的吸附能力。考虑到实验所用的纯水pH在6~7之间,因此不调节水样的pH。

#### 2.3.4 吸附时间

吸附时间是影响萃取效率的重要因素,考察了萃取时间分别为1、3、4、5、6 min时的萃取效果,如图5d所示。杀菌剂的萃取效率随萃取时间的增加逐渐升高,在吸附时间到达4 min后,继续延长萃取时间萃取效果无明显提升。因此实验选择的萃取时间为4 min。

#### 2.3.5 洗脱液类型

不同种类的洗脱液对洗脱效率有较大影响,比较了甲醇和乙腈两种洗脱溶液的洗脱效果。使用乙腈进行洗脱时,MBC和TBZ的回收率显著高于甲醇,FBZ和IPT的回收率略高于甲醇,因此选用乙腈作为洗脱溶液。为进一步提高洗脱效果,在洗脱溶液乙腈中加入氨水,以破坏材料与杀菌剂之间的作用力。考察了洗脱剂中氨水的体积分数分别为0、2%、3%、4%和5%时的萃取效果。结果如图5e所示,随着氨水含量从0增加到3%, TBZ、MBC和IPT的萃取效率逐渐提高,而从3%提高到5%后萃取效率呈降低的趋势;FBZ的萃取效率则是随着氨水含量的增加而降低。因此后续实验中在洗脱液乙腈中加入3%的氨水。

#### 2.3.6 洗脱液体积

洗脱液的体积也是影响洗脱效率的因素之一,对洗脱液用量分别为0.5、1、1.5和2 mL时的萃取效果进行评估。如图5f,洗脱液的体积由0.5 mL增加至1.5 mL时,萃取效率提高,从1.5 mL逐渐增加至2 mL,萃取效率趋于稳定,因此选择洗脱液体积为1.5 mL。

#### 2.3.7 洗脱时间

洗脱时间同样影响洗脱效率,实验考察了洗脱时间分别为0.5、1、2、3和4 min时的萃取效果。图5g结果表明,洗脱时间从0.5 min增加到1 min,萃取效率明显提高,而继续延长洗脱时间,萃取效率无显著增加,1 min足以充分解吸。因此洗脱时间选择1 min。

#### 2.3.8 NaCl含量

NaCl的加入会影响溶液的离子强度以及目标物的扩散速率,为了考察NaCl含量对萃取效果的影响,调节水样中NaCl的质量分数分别为0、0.5%、3%和10%。如图5h所示,随着NaCl含量的增加,萃取效率明显呈降低的趋势,可能是NaCl在水溶液中解离产生自由Na^+^,在Fe_3_O_4_@TpBD表面与杀菌剂分子竞争吸附位点^[25]^,阻碍了目标物与Fe_3_O_4_@TpBD之间的静电相互作用。另外NaCl的加入增加了水的黏度,降低了目标物在水中的传质效率。考虑到本方法主要用于自来水和地表水样品的测定,因此无需调节样品的离子强度。

### 2.4 方法学验证

#### 2.4.1 线性范围、检出限和定量限

对杀菌剂质量浓度分别为3、15、60、150、600、1200 ng/L的模拟水样进行检测,以质量浓度为横坐标、回收率为纵坐标绘制标准曲线。如表3所示,4种杀菌剂在3~1200 ng/L范围内具有良好的线性关系,相关系数(*r*^2^)均大于0.998,检出限(LOD, *S/N*=3)和定量限(LOQ, *S/N*=10)分别在0.06~0.28 ng/L和0.20~0.92 ng/L之间。

**表3:** 4种杀菌剂的线性方程、相关系数、线性范围、LOD和LOQ

Analyte	Linear equation	r^2^	Linear range/ (ng/L)	LOD/ (ng/L)	LOQ/ (ng/L)
TBZ	y=231.41x-809.83	0.9991	3-1200	0.07	0.23
FBZ	y=95.48x+16.98	0.9987	3-1200	0.28	0.92
MBC	y=219.86x-555.99	0.9997	3-1200	0.14	0.47
IPT	y=974.98x+675.58	0.9996	3-1200	0.06	0.20

*y*: peak area; *x*: mass concentration, ng/L.

#### 2.4.2 回收率和精密度

在空白水样中添加不同体积的标准储备液,配制成杀菌剂质量浓度分别为15、150、600 ng/L的加标水样,进行加标回收试验。每个浓度点1 d内测定6个平行样考察日内精密度,连续测定6 d考察日间精密度。如表4所示,4种杀菌剂的加标回收率为79.0%~107.5%,日内和日间精密度分别为2.8%~10.0%和4.4%~15.7%。

**表4:** 4种杀菌剂的回收率和精密度(*n*=6)

Analyte	Spiked/ (ng/L)	Recovery/ %	RSDs(n=6)	
			Intra-day/%	Inter-day/%
TBZ	15	106.3	8.7	10.8
	150	103.1	6.4	4.7
	600	93.0	5.4	4.4
FBZ	15	97.3	2.8	5.6
	150	90.3	8.6	6.6
	600	106.8	5.2	8.4
MBC	15	102.9	10.0	11.7
	150	93.4	9.2	12.1
	600	91.5	6.6	15.7
IPT	15	79.0	9.9	9.8
	150	107.5	6.7	9.7
	600	105.8	7.1	7.8

### 2.5 实际样品分析

为验证该方法在实际应用中的可行性,利用所建立的方法对实验室自来水和水库水进行分析检测。如表5所示,自来水中没有检测到杀菌剂的存在;在水库水中检测到微量的多菌灵,检出质量浓度为27.5 ng/L,低于欧洲水框架指令对天然水中的限值规定(0.1 μg/L)。自来水和地表水的加标回收率分别为85.0%~117.1%和77.1%~119.1%,表明该方法适用于环境水样中杀菌剂的富集和检测。

**表5:** 实际水样中4种杀菌剂的分析结果(*n*=3)

Analyte	Spiked/ (ng/L)	Tap water			Reservoir water	
		Found/ (ng/L)	Recovery (RSD)/%		Found/ (ng/L)	Recovery (RSD)/%
TBZ	0	ND			ND	
	15	14.9	99.5 (11.8)		13.5	89.8 (3.7)
	150	130.2	86.8 (3.4)		115.7	77.1 (10.1)
	600	651.7	108.6 (3.6)		529.3	88.2 (11.0)
FBZ	0	ND			ND	
	15	15.5	103.2 (11.2)		13.1	87.5 (10.8)
	150	158.2	105.5 (6.5)		124.1	82.8 (9.5)
	600	702.6	117.1 (2.7)		570.2	95.0 (5.1)
MBC	0	ND			27.5	
	15	13.1	87.5 (8.9)		17.8	119.1 (5.1)
	150	135.6	90.4 (16.7)		132.7	88.5 (2.6)
	600	579.5	96.0 (11.5)		529.2	88.2 (3.6)
IPT	0	ND			ND	
	15	12.7	85.0 (13.4)		12.0	80.1 (8.9)
	150	146.6	97.7 (4.2)		120.0	80.0 (3.7)
	600	660.3	110.0 (9.7)		550.4	91.7 (15.1)

ND: not detected.

### 2.6 与文献方法对比

将建立的分析方法与文献报道的杀菌剂的分析方法进行比较,如表6所示。本方法采用涡旋辅助MSPE的样品前处理方式,通过外部磁铁将Fe_3_O_4_@TpBD从水中分离,操作简单且快速,省略了氮吹步骤,大幅缩减了样品前处理时间,再结合超高效液相色谱-串联质谱进行检测,获得了最低的方法检出限。

**表6:** 本方法与文献报道的杀菌剂分析方法比较

Analysis method	Sample pretreatment materials	Analytes	Matrices	Pretreatment time/min	LODs	Ref.
MSPE-HPLC-UV	Fe_3_O_4_@COF	TBZ, MBC, ABZ, 2-ABZ, ABZSO	fruits and juice	>26	2.5-2.9 μg/L	[9]
SPME-HPLC-UV	MWCNTs-COOH	TBZ, MBC, TM	river and wastewater	>35	0.3-1.5 μg/L	[7]
MSPE-HPLC-UV	Ni/CTF-SO_3_H	TBZ, MBC	fruits, vegetables and juices	>5	1.23-7.05 μg/kg	[3]
MISPE-HPLC-FLD	molecularly imprinted monolithic column	MBC, TBZ, FBZ	citrus	>60	0.03-9.68 μg/L	[26]
SPE-HPLC-FLD	SiO_2_@NiO	MBC, TBZ	fruit and vegetable	>5	2.9-7.5 μg/kg	[27]
SPE-UHPLC-MS/MS	HLB cartridge	IPT	field paddy water	>50	0.0015 μg/L	[8]
MSPE-HPLC-FLD	benzenesulfonic acid- modified Fe_3_O_4_@SiO_2_	MBC, TBZ	fruits and juice	>5	0.14-0.55 μg/kg	[28]
MSPE-UHPLC-MS/MS	Fe_3_O_4_@TpBD	TBZ, MBC, FBZ, IPT	tap and reservoir water	5	0.06-0.28 ng/L	this work

HPLC-UV: high performance liquid chromatography-ultraviolet detection; MISPE-HPLC-FLD: molecularly imprinted solid phase extraction-high performance liquid chromatography-fluorescence detection. TM: thiophanate-methyl; 2-ABZ: 2-aminobenzimidazole; ABZ: albendazole; ABZSO: albendazole sulfoxide.

## 3 结论

本文制备了磁性共价有机骨架材料Fe_3_O_4_@TpBD作为萃取吸附剂,利用材料与目标物之间的*π-π*共轭和氢键作用进行吸附,建立了一种基于涡旋辅助MSPE结合UHPLC-MS/MS检测环境水样中4种杀菌剂的分析方法。该方法具有良好的灵敏度、精密度和准确度,操作简单,耗时短,满足实际样品的检测要求,为环境水体中杀菌剂的研究分析提供了理论基础。Fe_3_O_4_@TpBD材料合成简单、成本较低,是一种优良的吸附材料,今后可拓展基于该材料的样品前处理技术分析环境水体中其他污染物的应用。

## References

[1] Li Q S, Yu F, Cao G Z, et al. Environmental Protection, 2021, 49(10): 13

[2] Wen Y, Li J, Yang F, et al. Talanta, 2013, 106: 119 23598103 10.1016/j.talanta.2012.12.011

[3] Zhao W, Wang X, Guo J, et al. J Chromatogr A, 2020, 1618: 460847 31928768 10.1016/j.chroma.2019.460847

[4] SantaladchaIyakit Y, Phiroonsoontorn N, Sillapatiwat C, et al. J Brazil Chem Soc, 2015, 26(10): 2014

[5] Kakimoto Y, Takatori S, Okihashi M, et al. Food Anal Method, 2016, 9(12): 3345

[6] Zamora O, Paniagua E E, Cacho C, et al. Anal Bioanal Chem, 2009, 393(6/7): 1745 19184593 10.1007/s00216-009-2631-1

[7] Ji Y, Liu X, Jiang X, et al. Chromatographia, 2009, 70(5/6): 753

[8] Zaidon S Z, Ho Y B, Hamsan H, et al. Microchem J, 2019, 145: 614

[9] Li S, Liang Q, Ahmed S A H, et al. Food Anal Method, 2020, 13(5): 1111.

[10] Ji X F, Li S, Wu G G, et al. Chinese Journal of Chromatography, 2021, 39(8): 896 34212590 10.3724/SP.J.1123.2021.01006 PMC9404032

[11] Ma J P, Jiang L H, Wu G G, et al. J Chromatogr A, 2016, 1466: 12 27590086 10.1016/j.chroma.2016.08.065

[12] Ma J P, Wu G G, Li S, et al. J Chromatogr A, 2018, 1553: 57 29685336 10.1016/j.chroma.2018.04.034

[13] Li S, Ma J P, Wu G G, et al. J Hazard Mater, 2022, 424(Pt D): 127687 34776299 10.1016/j.jhazmat.2021.127687

[14] Ma J P, Yao Z D, Hou L W, et al. Talanta, 2016, 161: 686 27769466 10.1016/j.talanta.2016.09.035

[15] Cote A P, Benin A I, Ockwig N W, et al. Science, 2005, 310(5751): 1166 16293756 10.1126/science.1120411

[16] Zhang Y, Song Y, Wu J, et al. Chem Commun, 2019, 55(26): 3745 10.1039/c9cc00384c 30860237

[17] Wang L, Deng M, Xu H, et al. ACS Appl Mater Inter, 2020, 12(33): 37619 10.1021/acsami.0c11463 32814408

[18] Romero V, Fernandes S P S, Ková Ř P, et al. Micropor Mesopor Mat, 2020, 307: 110523

[19] Xu G, Dong X, Hou L, et al. Anal Chim Acta, 2020, 1126: 82 32736728 10.1016/j.aca.2020.05.071

[20] Wu M, Chen G, Ma J, et al. Talanta, 2016, 161: 350 27769417 10.1016/j.talanta.2016.08.041

[21] Li N, Wu D, Hu N, et al. J Agric Food Chem, 2018, 66(13): 3572 29554797 10.1021/acs.jafc.8b00869

[22] Tan J, Namuangruk S, Kong W, et al. Angew Chem Int Edit, 2016, 55(45): 13979 10.1002/anie.201606155 27709769

[23] Pang Y H, Yue Q, Huang Y Y, et al. Talanta, 2020, 206: 120194 31514904 10.1016/j.talanta.2019.120194

[24] Li N, Wu D, Liu J, et al. Microchem J, 2018, 143: 350

[25] Jeong Y, Schaffer A, Smith K. Chemosphere, 2017, 174: 297 28183055 10.1016/j.chemosphere.2017.01.116

[26] Liang G, Guo X, Tan X, et al. Microchem J, 2019, 146: 1285

[27] Yu Q W, Sun H, Wang K, et al. Food Anal Method, 2017, 10(8): 2892

[28] Deng X, Chen X, Lin K, et al. Food Anal Method, 2013, 6(6): 1576

